# Impact of alanyl-tRNA synthetase editing deficiency in yeast

**DOI:** 10.1093/nar/gkab766

**Published:** 2021-09-09

**Authors:** Hong Zhang, Jiang Wu, Zhihui Lyu, Jiqiang Ling

**Affiliations:** Department of Cell Biology and Molecular Genetics, The University of Maryland, College Park, MD 20742, USA; Department of Microbiology and Molecular Genetics, McGovern Medical School, University of Texas Health Science Center, Houston, TX 77030, USA; Department of Cell Biology and Molecular Genetics, The University of Maryland, College Park, MD 20742, USA; Department of Cell Biology and Molecular Genetics, The University of Maryland, College Park, MD 20742, USA

## Abstract

Aminoacyl-tRNA synthetases (aaRSs) are essential enzymes that provide the ribosome with aminoacyl-tRNA substrates for protein synthesis. Mutations in aaRSs lead to various neurological disorders in humans. Many aaRSs utilize editing to prevent error propagation during translation. Editing defects in alanyl-tRNA synthetase (AlaRS) cause neurodegeneration and cardioproteinopathy in mice and are associated with microcephaly in human patients. The cellular impact of AlaRS editing deficiency in eukaryotes remains unclear. Here we use yeast as a model organism to systematically investigate the physiological role of AlaRS editing. Our RNA sequencing and quantitative proteomics results reveal that AlaRS editing defects surprisingly activate the general amino acid control pathway and attenuate the heatshock response. We have confirmed these results with reporter and growth assays. In addition, AlaRS editing defects downregulate carbon metabolism and attenuate protein synthesis. Supplying yeast cells with extra carbon source partially rescues the heat sensitivity caused by AlaRS editing deficiency. These findings are in stark contrast with the cellular effects caused by editing deficiency in other aaRSs. Our study therefore highlights the idiosyncratic role of AlaRS editing compared with other aaRSs and provides a model for the physiological impact caused by the lack of AlaRS editing.

## INTRODUCTION

Accurate transfer of genetic information from DNA to RNA to protein requires precise processes in DNA replication (1,2), transcription (3) and translation (4–6). A central step to maintain such fidelity is the accurate pairing of amino acids with corresponding transfer RNAs (tRNAs) by aminoacyl-tRNA synthetases (aaRSs). Given the similarity between some amino acids, approximately half of the 20 aaRSs utilize an editing activity to proofread misactivated (pre-transfer editing) or misacylated amino acids (post-transfer editing) (7–10). In addition, free standing trans-editing factors provide further proofreading to remove incorrect aminoacyl-tRNAs (aa-tRNAs) (11–14). Failure of proofreading during aminoacylation results in amino acid misincorporation in the proteome and a substantially increase in the errors during protein synthesis (15–17).

The editing function of aaRSs is mostly conserved from bacteria to higher eukaryotes throughout evolution. It is therefore puzzling that disrupting editing of some aaRSs appears to be well tolerated in bacteria and eukaryotes. For instance, ablating the editing function of threonyl-tRNA synthetase (ThrRS) has little effect on the growth of *Escherichia coli* (17,18). Editing deficiency in phenylalanyl-tRNA synthetase (PheRS) also shows no growth effect in *E. coli* unless an oxidized form of tyrosine (*meta*-tyrosine) accumulates (19). In yeast, leucyl-tRNA synthetase (LeuRS) editing defects does not affect growth under normal conditions (20). In contrast, mild editing defects in alanyl-tRNA synthetase (AlaRS) lead to neurodegeneration (loss of Purkinje cells) and cardiac proteinopathy in mice, and a more severe AlaRS editing defect is embryonic lethal (21,22). In humans, an AlaRS mutation that causes both aminoacylation and editing defects has been associated with patients suffering early-childhood neurodegeneration and progressive microcephaly (23). AlaRS editing deficiency appears to selectively damage neuron cells and cardiomyocytes. Although elevated levels of protein ubiquitination and aggregation have been observed in these cells of the editing-defective mice (21,22), the overall cellular impact of AlaRS editing remains elusive. In this study, we use *Saccharomyces cerevisiae* as a model organism to compare the cellular impact of AlaRS editing and aminoacylation defects, respectively. Combining RNA sequencing, proteomics, genetic, and biochemical analyses, we show that editing deficiency in AlaRS activate the general amino acid control (GAAC) pathway and causes severe growth defects under heat stress. We further show that the heat sensitivity caused by AlaRS editing deficiency is partially due to impaired carbon metabolism, which may lead to a weakened heatshock response.

## MATERIALS AND METHODS

### Plasmids and strains

All *S. cerevisiae* strains used here were derivatives of BY4741. *Escherichia coli* DH5α grown in LB medium were used for cloning. *ALA1* (encoding AlaRS) point mutants C719A and G906D were obtained by plasmid shuffling. Briefly, pRS315-*ALA1*-C719A and pRS315-*ALA1*-G906D were transformed into BY4741 *ala1*Δ carrying pRS316-*ALA1*-WT using the lithium acetate method, and the *URA3* pRS316 plasmid was cured by counter selection on 5-fluoroorotic acid containing synthetic defined (SD)–Leu agar plates.

To create the *gcn2*Δ, *gcn4*Δ, *alaX*Δ, *ltn1*Δ, *rqc1*Δ, *rqc2*Δ, *eap1*Δ and *caf20*Δ mutants, the coding regions were replaced with *URA3* or *HIS2* gene. Cells were grown in either YPD (1% yeast extract, 2% peptone and 2% glucose) media or SD dropout medium (0.17% yeast nitrogen base, 0.5% ammonium sulfate, 2% glucose and 0.14% amino acid dropout mix without His, Leu or Ura). All strains and plasmids are listed in Supplementary Table S4 and all the primers used are listed in Supplementary Table S5.

### Aminoacylation and editing assays *in vitro*

N-terminal His_6_-tagged yeast and human AlaRS variants were expressed and purified from *E. coli* as described (23). Aminoacylation and editing assays were performed as described (23,24) with slight modifications. Aminoacylation activity was determined using 0.3–1 μM AlaRS, 20 mg/ml total yeast tRNAs (Roche), 4 mM ATP and 40 μM [^14^C] Ala (73 cpm/pmol) in aminoacylation buffer (100 mM HEPES–NaOH, pH 7.2, 30 mM KCl and 10 mM MgCl_2_) at 37°C. Aliquots were spotted on 3MM Whatman paper discs presoaked with 5% trichloroacedic acid (TCA), washed three times with 5% TCA, dried, and their radioactivity level was measured in the scintillation counter.

The substrate of editing assays [^14^C] Ser-tRNA^Ala^ was prepared by charging 20 mg/ml total yeast tRNAs with 100 μM [^14^C] Ser (67 cpm/pmol) by the editing-defective human AlaRS 723A mutant in aminoacylation buffer at 37°C for 10 min. 1 μM yeast AlaRS was then added to hydrolyze Ser-tRNA. At each time point, an aliquot was spotted on 3MM Whatman paper discs presoaked with 5% TCA, washed three times with 5% TCA, dried, and scintillation counted.

### Temperature sensitivity analysis

Liquid culture growth assays were tested using Synergy HT microplate reader (BioTek, USA) at 30 or 37°C. WT, C719A, G906D and their *gcn2*Δ, *gcn4*Δ and *alaX*Δ derivative strains were grown in YPD, and tRNA overexpression strains were grown in SD–Leu. For agar plating assay, cells were grown to log phase in liquid media at 30°C. 5- or 10-fold serial dilutions were spotted on YPD or SD - Leu agar plates and incubated both at 30°C and 37°C for 2–4 days before imaging.

### β-Lactamase assay

To determine Ser misincorporation at Ala codons, WT, C719A, G906D cells expressing the WT (pJL-*bla*) or S68A mutant (pJL-*bla*-S68A-GCC) β-lactamase or carrying the control plasmid pJD1212 were grown in SD–Ura to log phase. The same OD_600_ (0.9) of cells were collected, washed and resuspended in 200 μl lysis buffer (PBS pH 7.0, 1 mM phenylmethylsulfonyl fluoride (PMSF), 10 mM dithiothreitol (DTT), 100× protein inhibitor cocktail and 3 mg/ml zymolyase) and incubated at room temperature for 40 min. β-Lactamase activity (25) was determined using a 100 μl reaction mixture containing PBS pH7.0, 100 μM nitrocefin and 10 μl cell lysate. β-Lactamase with Ser at position 68 converts nitrocefin to a product with absorption at 486 nm, which was measured using the Synergy HT microplate reader.

### β-Galactosidase assay

To determine the translational regulation of *GCN4*, WT, C719A or G906D yeast cells with P*gcn4-lacZ* reporters pJD821, pJD822 and pJD823 were grown in SD–Ura medium to log phase at 30°C. 700 μl culture were collected and resuspended in 700 μl Z-buffer (60 mM Na_2_HPO_4_, 40 mM NaH_2_PO_4_, 10 mM KCl and 1 mM MgSO_4_), and cell density was measured by OD_600_. Cells were lysed by adding 100 μl chloroform and 50 μl 0.1% SDS and vortexing for 15 s. Reaction was initiated by addition of 0.2 ml pre-warmed ONPG (o-nitrophenyl-β-galactoside, 4 mg/ml in Z-buffer); after yellow color developed, the reaction was terminated by adding 500 μl 1 M Na_2_CO_3_. Cell debris was removed by centrifugation, and OD_420_ of the supernatant was determined. β-Galactosidase activity was calculated according to the following equation: Miller units = 1000 × OD_420_/(*T* × *V* × OD_600_) (26,27).

### Isolation of aggregated proteins

Aggregated proteins were prepared as previously described (28). Briefly, 1 ml log-phase yeast cells were washed and harvested, and the pellets were resuspended in 200 μl lysis buffer (PBS pH7.0, 10 mM DTT, 1 mM PMSF, 100× protease inhibitor cocktail, and 3 mg/ml zymolyase), then incubated at room temperature for 40 min with 20 min intervals for vortexing. After brief sonication, the samples were centrifuged for 20 min at 200 g at 4°C. Supernatants were then adjusted to the same protein concentration using Bradford method, and the aggregates were collected by centrifuging at 16 000 g for 20 min. After removal of the supernatant, aggregated proteins were washed twice with 2% NP-40 in PBS, sonicated and centrifuged at 16 000 g for 20 min. The aggregated proteins were then washed twice with PBS and sonicated. The aggregates and equal amounts of total proteins were then boiled in SDS loading buffer and separated on 12% SDS-PAGE gels, and finally analyzed by Coomassie brilliant blue staining.

### Determination of heatshock response activity

The live-cell luciferase assay was performed essentially as described (29). WT, C719A and G906D strains containing the heatshock response reporter pHSE-lucCP^+^ and the control salt inducible reporter pGre2-lucCP^+^ were grown in SD–His at 30°C to log phase and adjusted to the same concentration. Prior to checking the heat-induced luminescence, pGre2-lucCP^+^ harboring strains were incubated with 0.4 M NaCl, and all strains were shaken at 30°C for 1 h. 500 μM luciferin was added to all strains in a 96-well plate, which was immediately placed in a 37°C microplate reader to record the luminescence intensity.

The heatshock response reporter pHSE-GFP was transformed into the WT, C719A, G906D strains and the resulting transformants were grown in SD–Ura medium to log phase. To examine heat-induced GFP expression, strains were adjusted to the same OD_600_ and placed in a 37°C microplate reader for fluorescence measurement.

### Fluorescence microscopy

The heat-induced aggregation reporter pHSP104-RFP (30) was transformed into the WT, C719A and G906D strains, and the resulting transformants were grown in SD–Ura medium to log phase. 1 μl yeast cells were pipetted onto a 2 μl 1% agarose PBS pad on a 15-well slide. Cells were visualized on a BZ-X810 inverted microscope (Keyence).

### Ribosome quality control analysis

Ribosome quality control reporter was tested as described (31). The WT, C719A, and G906D strains and their *ltn1*Δ, *rqc1*Δ and *rqc2*Δ strains carrying the pTDH3-GFP-R12-RFP were grown in SD–Ura. Cell pellets were resuspended in 200 μl lysis buffer (PBS pH7.0, 10 mM DTT, 1 mM PMSF, 100× protease inhibitor cocktail, and 3 mg/ml zymolyase), and incubated at room temperature for 30 min with 15 min intervals for vortexing. After sonication and centrifugation (16 000 g for 5 min), supernatant proteins were separated by 12% SDS-PAGE and transferred to nitrocellulose membranes. Next, standard western blotting procedure was followed to detect the GFP levels. The mouse anti-GFP first antibody (Invitrogen) with 1:1000 dilution and goat anti- mouse IgG-HRP secondary antibody (Invitrogen) with 1:5000 dilution were used. Nitrocellulose membranes were treated with ECL chemiluminescent substrate reagents (Bio-Rad) and visualized using a ChemiDoc Imaging System (Bio-Rad).

### Protein synthesis assay

To quantify the global protein synthesis rate, log-phase WT, C719A and G906D cells were adjusted to the same OD_600_, washed twice with SD–methionine (Met) medium, and then the protocol of Click-iT^®^ HPG Alexa Fluor^®^ 594 protein synthesis assay kit (Thermo Fisher) was followed. This method is based on the co-translational incorporation of HPG (l-homopropargylglycine), which is an analog of methionine containing an alkyne moiety. Cell pellets were incubated with 100 μl SD - Met containing 50 μM HPG at 30 or 37°C for 1 h. Following fixation and fluorophore labeling, the *A*_600_ and the fluorescence signal intensity were determined using a Synergy HT microplate reader.

### Transcriptome analysis

WT, C719A, and G906D cells were grown in YPD at 30°C to log phase, and incubated at 37°C for additional 0, 1 or 2 h. Total RNA was prepared using an RNA extraction kit (Qiagen). Library construction and Illumina sequencing were performed by Novogene.

### Label-free quantitative proteomics analysis

Label-free quantitative proteomics was performed by Creative Proteomics (New York, USA). Three biological replicate samples of each strain were prepared and analyzed. Log-phase WT, C719A and G906D cells were incubated at 30 or 37°C for 2 h. Cell pellets from 2 ml cultures were fast frozen in liquid nitrogen. Protein extraction, trypsin digestion, and LC–MS/MS analysis were performed by Creative Proteomics (New York, USA). The resulting MS/MS data were processed using MaxQuant software. Gene expression with 1.5-fold difference of label-free quantitation (LFQ) intensity and *P* value <0.05 was considered significant. The identified differentially expressed proteins were enriched by Gene Ontology (GO) annotation and KEGG pathway analyses using metascape (32).

## RESULTS

### AlaRS editing deficiency causes serine misincorporation and heat sensitivity

Alanyl-tRNA synthetase misacylates serine (Ser) and glycine onto tRNA^Ala^ and uses both a *cis*-editing site and *trans*-editing factors to hydrolyze the misacylated tRNAs (14,34–37). To evaluate the cellular effects of editing and aminoacylation defects, we constructed haploid yeast strains carrying the wild type (WT), C719A or G906D AlaRS variants on a single-copy plasmid in the AlaRS (*ALA1*) deletion background. The C719A mutation in yeast AlaRS is equivalent to C666A in *E. coli* and C723A in humans that selectively inactivate editing but not aminoacylation (18,23,34) (Supplementary Figure S1). The G906D mutation in yeast is equivalent to G913D in humans, which is identified in microcephaly patients and decreases the aminoacylation but not editing efficiency (23). Our *in vitro* experiments using recombinant yeast AlaRS variants also confirm that C719A and G906D mutations cause editing and aminoacylation defects, respectively (Supplementary Figure S2). We additionally attempted to construct a mutant strain carrying a truncated AlaRS identified in microcephaly patients with both aminoacylation and editing defects (23) but were unsuccessful, suggesting that this mutant AlaRS is not able to support yeast growth.

**Figure 1.**
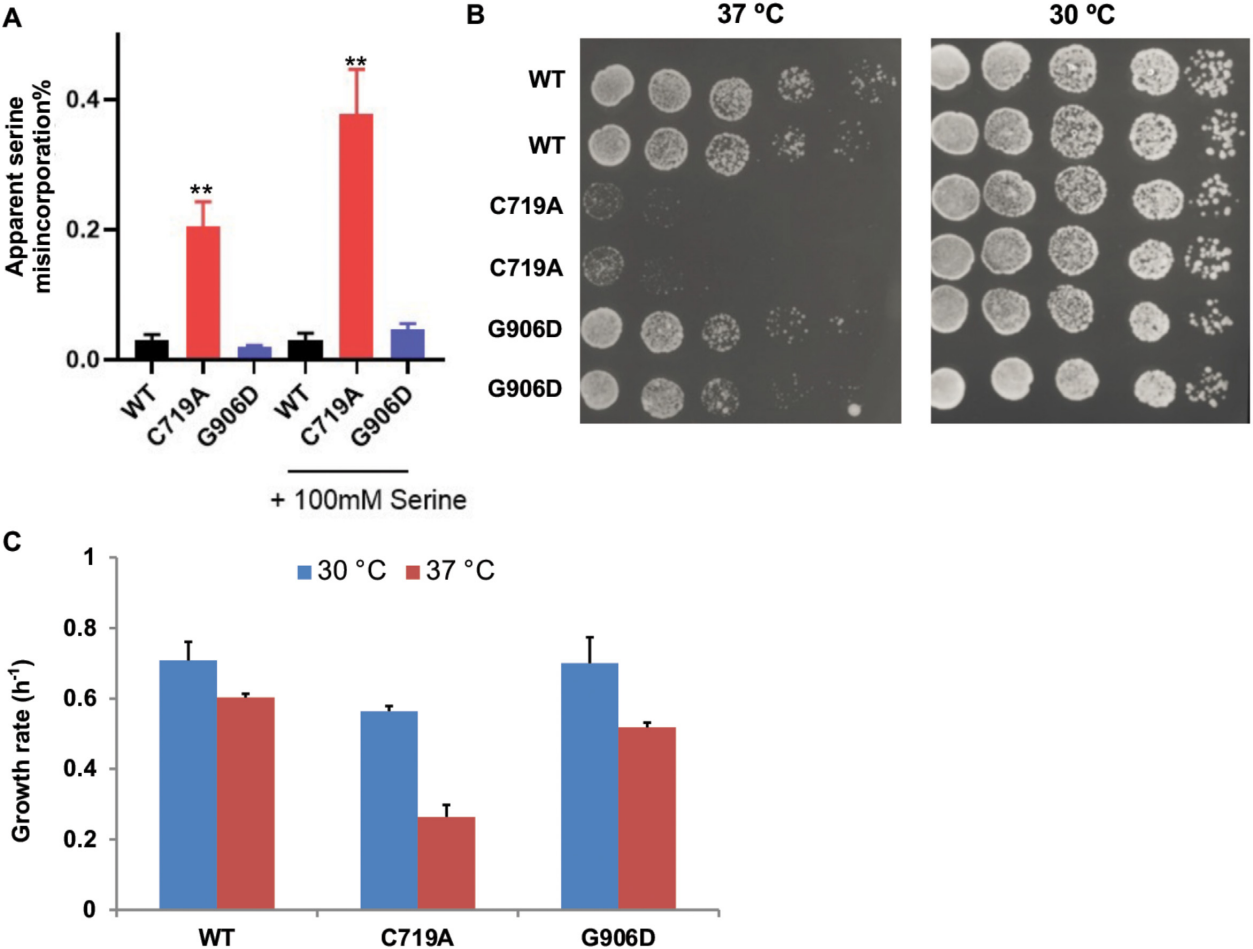
C719A mutation in yeast AlaRS causes serine misincorporation and heat sensitivity. (**A**) Ser misincorporation rates in the WT, C719A, and G906D yeast strains. Cells were grown in SD–Ura with or without additional Ser (100 mM). The rates were calculated as the activity ratio of the S68A β-lactamase variant over the WT β-lactamase. Misincorporation of Ser at Ala codons results in active β-lactamase for the S68A variant. The C719A mutant displayed a significant increase in Ser misincorporation at Ala codons. (**B**) Yeast cells were grown to log phase and spotted on YPD agar plates with 5-fold dilutions. The plates were incubated at 30 or 37°C for 2 days before imaging. (**C**) Growth rates of yeast strains in YPD liquid culture at 30 or 37°C. These results are the average or representatives of at least three biological replicates. Error bars represent one standard deviation (SD) from the mean. The *P* value is determined using the unpaired *t* test. ** *P* < 0.01.

To evaluate mistranslation rates resulting from AlaRS editing deficiency, we transformed a β-lactamase gene with a critical active-site Ser (S68) codon changed to an alanine (Ala) codon and quantitated the enzyme activity in yeast. Misincorporation of Ser into the A68 position would yield active β-lactamase proteins (16,18,25). The C719A mutation increased Ser misincorporation 7-fold from 0.03% to 0.2%, whereas the G906D mutation did not affect Ser misincorporation (Figure 1A). Additional Ser in the media further increased the Ser misincorporation rate to 0.4% in the C719A strain. These results confirm that the C719A mutation causes an AlaRS editing defect in yeast and promotes mistranslation despite the presence of the trans*-*editing factor AlaX.

**Figure 2.**
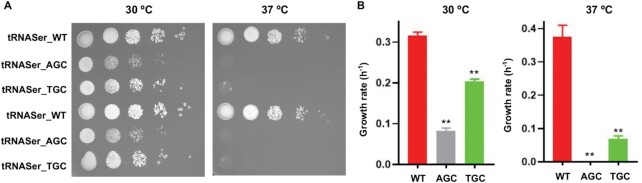
Expressing tRNA^Ser^ variants reading Ala codons impair yeast growth. (**A**) WT yeast carrying pRS315 tRNA^Ser^ variants were grown in SD–Leu medium to log phase and spotted on SD–Leu plates with 10-fold dilutions. The plates were incubated at 30 or 37°C for 4 days before imaging. (**B**) Growth rates of yeast strains in SD–Leu liquid culture at 30 or 37°C. These results are the average or representatives of at least three biological replicates. Error bars represent standard errors. The *P* value is determined using the unpaired *t* test. ** *P* < 0.01.

We next tested growth of the resulting strains at various temperatures. Both the C719A and G906D mutant strains grew as well as the WT at 30°C, but the C719A strain displayed severe growth defects both on the yeast peptone dextrose (YPD) agar plate and in YPD liquid culture at 37°C, suggesting that AlaRS editing deficiency leads to increased sensitivity to heat (Figure 1B, C and Supplementary Figure S3). To test whether such heat sensitivity is due to Ser mistranslation, we expressed tRNA^Ser^ mutants with Ala anticodons (AGC and TGC) in WT yeast. Seryl-tRNA synthetase does not recognize the tRNA anticodon and therefore aminoacylates such tRNA variants with Ser, resulting in misreading of Ala codons as Ser. We found that similar to the AlaRS C719A mutation, tRNA^Ser^_AGC_ and tRNA^Ser^_TGC_ both caused severe growth defects at 37°C (Figure 2). In contrast, misincorporation of Ser at leucine (Leu) codons by tRNA^Ser^_CAG_ did not appear to cause heat sensitivity (Supplementary Figure S4). These results suggest that Ser misincorporation at Ala codons is particularly toxic under heat stress conditions.

### Transcriptomics and proteomics analyses of AlaRS mutants

**Figure 3.**
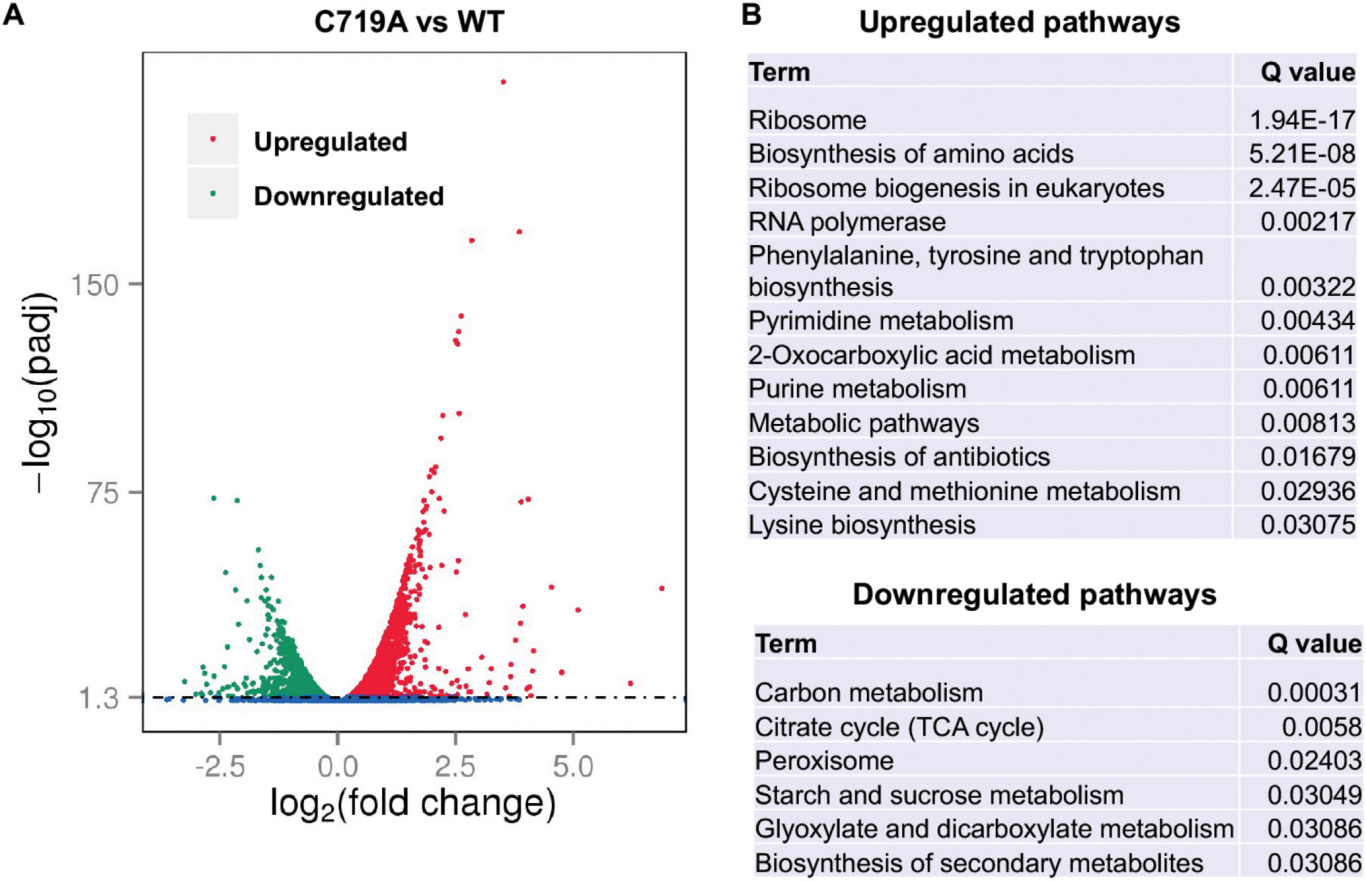
RNA sequencing comparison of C719A and WT yeast variants upon heat stress. Yeast cells were grown in YPD at 30°C to log phase and shifted to 37°C for 2 h before total RNA was prepared. (**A**) Volcano plot comparing C719A and WT. The Y-axis represent the adjusted *P* values. (**B**) Significantly enriched pathways in the C719A mutant compared with the WT. Three biological replicates were tested for each strain at each condition.

To gain a better understanding of how AlaRS editing and aminoacylation defects influence gene expression in yeast, we performed RNA sequencing analyses of the WT, C719A and G906D strains that were pre-grown at 30°C, before and after shifting to 37°C heat stress (Figure 2, Supplementary Figures S5 and S6, Table S1). Compared with the WT, both C719A and G906D strains showed significant upregulation in the ribosome and amino acid biogenesis pathways at 30 or 37°C. The most significantly downregulated pathways caused by the C719A mutation upon heat stress were carbon metabolism and the citrate cycle (Figure 3).

**Figure 4.**
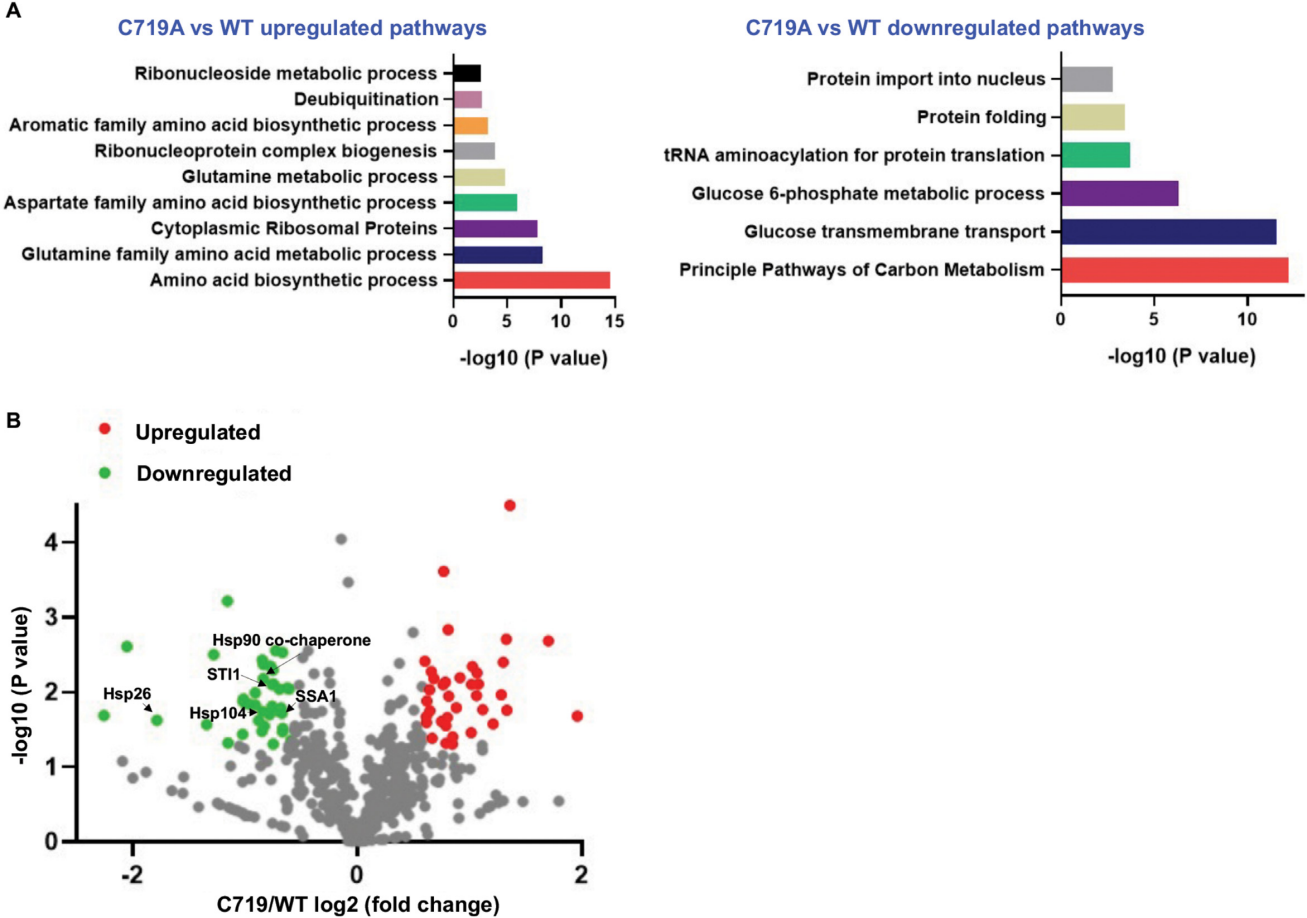
Quantitative proteomics of C719A yeast compared to WT upon heat stress. Yeast cells were grown in YPD at 30°C to log phase and shifted to 37°C for 2 h before rapid freezing and protein extraction. (**A**) Significantly enriched pathways in the C719A mutant compared with the WT. (**B**) Volcano plot comparing C719A and WT. The heatshock proteins that are significantly downregulated in the C719A mutant are indicated. Three biological replicates were tested for each strain at each condition.

While RNA sequencing analysis is a powerful tool to profile the transcriptome and understand regulation of gene expression, the mRNA levels may not always correlate with the protein levels due to translational and posttranslational regulations (38). We therefore performed additional label-free quantitative proteomics experiments. We found that like the RNA sequencing results, proteomics results revealed that upon heat stress ribosome and amino acid biogenesis were significantly upregulated in the C719A strain, and carbon metabolism was most significantly downregulated (Figure 4, Supplementary Tables S2 and S3). Interestingly, the protein folding pathway that include many heatshock proteins was also downregulated in the C719A strain. The proteome of the G906D strain was less consistent with the transcriptome (Supplementary Figure S7, Supplementary Tables S2 and S3). In particular, the amino acid biosynthesis proteins were less abundant in the G906D mutant compared with the WT, which was opposite to the mRNA levels, implicating additional translational and posttranslational control of this pathway.

### AlaRS editing deficiency activates the general amino acid control pathway

**Figure 5.**
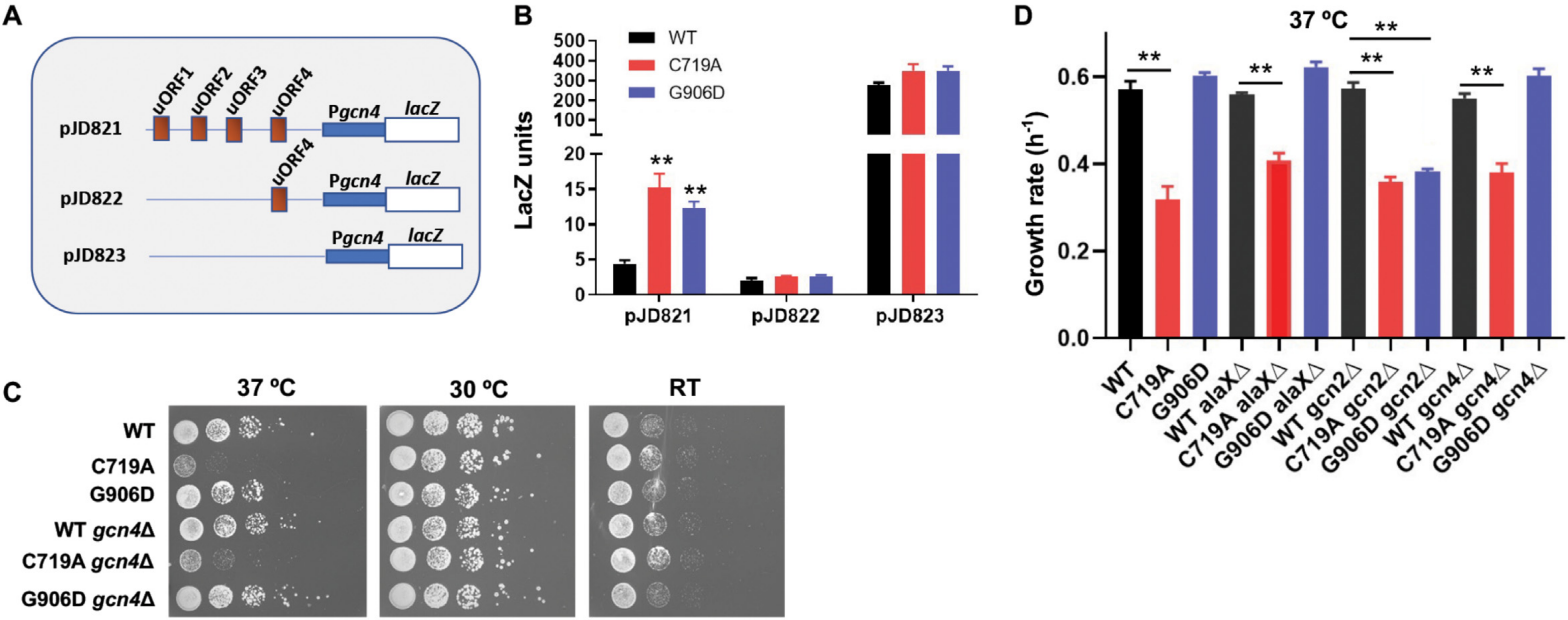
C719A and G906D AlaRS mutations activate the GCN4 GAAC pathway. (**A**) Plasmid constructs for the GCN4 reporter assay. The 5′ region of *GCN4*, including the regulatory uORFs and promoter, was fused with the *lacZ* gene. pJD821 contains all the regulatory elements, while pJD822 and pJD823 are negative and positive controls, respectively. (**B**) WT, C719A and G906D yeast cells carrying pJD plasmids were grown in SD–Ura medium to log phase at 30°C, and the LacZ activity was determined. (**C**) Yeast cells were grown to log phase and spotted on YPD agar plates with 10-fold dilutions. The plates were incubated at room temperature (RT), 30 or 37°C for 2 days before imaging. (**D**) Growth rates of yeast strains in YPD liquid culture at 37°C. These results are the average or representatives of at least three biological replicates. Error bars represent standard errors. The *P* value is determined using the unpaired *t* test. ** *P* < 0.01.

Our RNA sequencing and proteomics data both suggest that the C719A mutation causing AlaRS editing deficiency activates the amino acid biosynthesis pathway. In yeast, amino acid biosynthesis is controlled by the Gcn4p transcription factor, which regulates the GAAC pathway (39–42). Upon amino acid starvation, uncharged tRNAs accumulate and activate Gcn2p to phosphorylate eIF2A, which attenuates overall protein synthesis but selectively induces translation of *GCN4* via bypassing the open reading frame uORF4 (Figure 5A). GAAC thus provides a feedback mechanism to maintain amino acid homeostasis. To test the GAAC response, we used a *lacZ* reporter fused with the 5′ region (with or without uORFs) of *GCN4*. The plasmid pJD821 contains all the regulatory elements of *GCN4* translation, whereas pJD822 and pJD823 serve as constitutively repressed and derepressed controls, respectively (Figure 5A). We confirmed the RNA sequencing data that the GAAC pathway was indeed activated in the C719A and G906D strains compared with the WT (Figure 5B and Supplementary Figure S3C). To test whether GAAC was involved in the heat sensitivity of the editing-defective strain, we deleted the *GCN4* and *GCN2* genes separately in the WT, C719A and G906D strains. Neither *GCN4* or *GCN2* deletion rescued growth of the C719A strain at 37°C (Figure 5C and D), suggesting that the heat sensitivity phenotype caused by AlaRS editing deficiency is independent of the GAAC pathway.

### AlaRS editing deficiency attenuates translation

**Figure 6.**
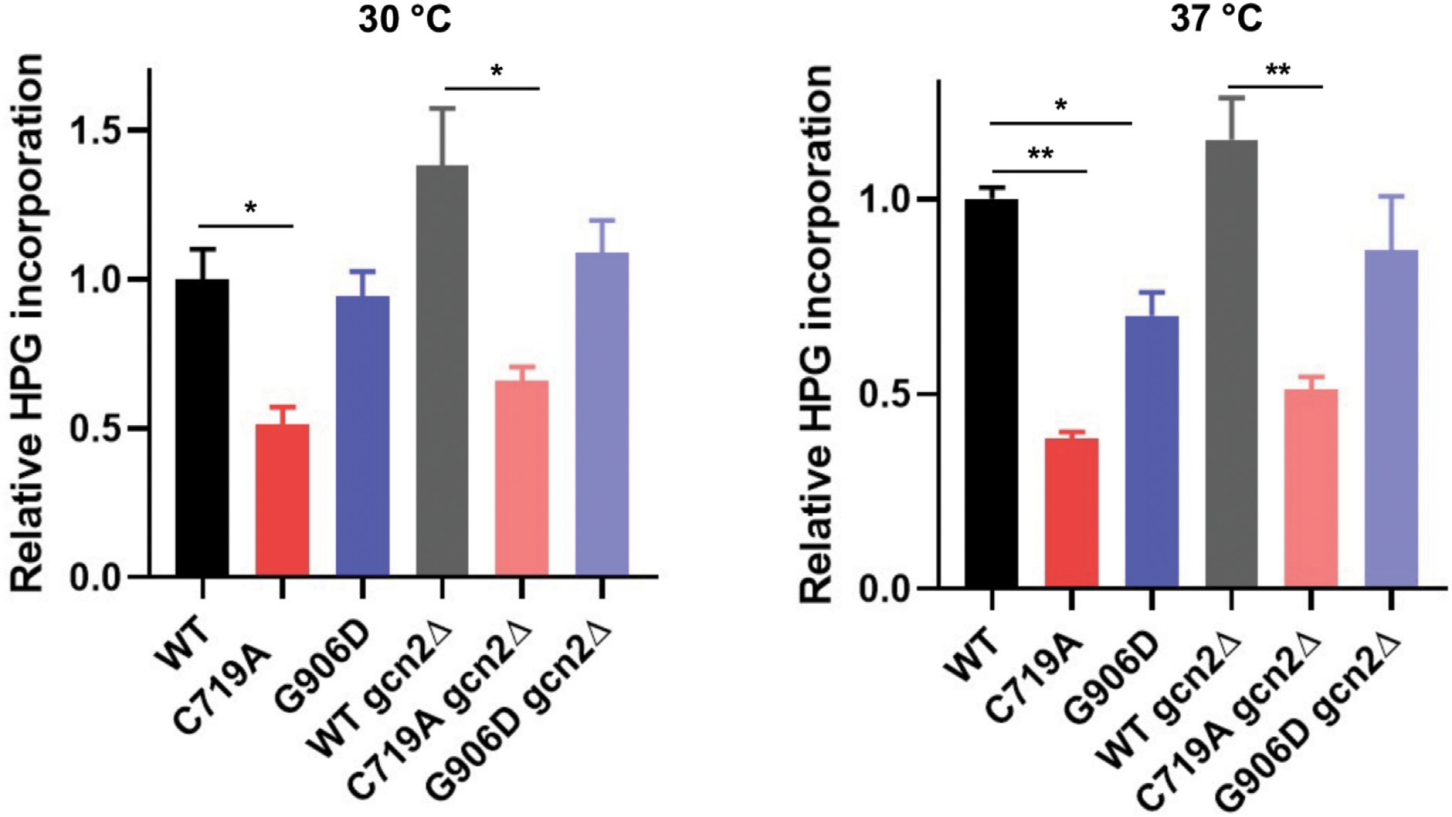
C719A AlaRS mutation attenuates translation. Cells were grown to log phase in YPD at 30°C and incubated with HPG in SD–Met medium at 30 or 37°C for 1 h. HPG was incorporated at Met codons in the proteome. The C719A mutant shows significant decreases in HPG incorporation, indicating a reduction in protein synthesis efficiency. These results are the average of at least three biological replicates. Error bars represent standard errors. The *P* value is determined using the unpaired *t* test. ** *P* < 0.01.

Phosphorylation of eIF2A by activated Gcn2p attenuates global translation initiation (39). We therefore measured the overall protein synthesis rate using a methionine analog homopropargylglycine (HPG). We found that HPG incorporation in the C719A strain was decreased over 50% at both 30 and 37°C compared with the WT, and the G906D mutant also had a lower translation efficiency at 37°C (Figure 6). Deleting *GCN2* did not restore protein synthesis in the AlaRS mutant strains. In yeast, there are two characterized binding proteins (Eap1p and Caf20p) that inhibit eIF4E during translation initiation (42), prompting us to test the role of Eap1p and Caf20p in repression of translation by the C719A AlaRS mutation. Deleting *CAF20*, but not *EAP1*, partially restored HPG incorporation in the C719A mutant (Supplementary Figure S8A and S8B). However, neither *Caf20*^–^ nor *Eap1*^–^ fully rescued heat sensitivity of the C719A strain (Supplementary Figure S8C).

### AlaRS editing deficiency dampens the heatshock response

**Figure 7.**
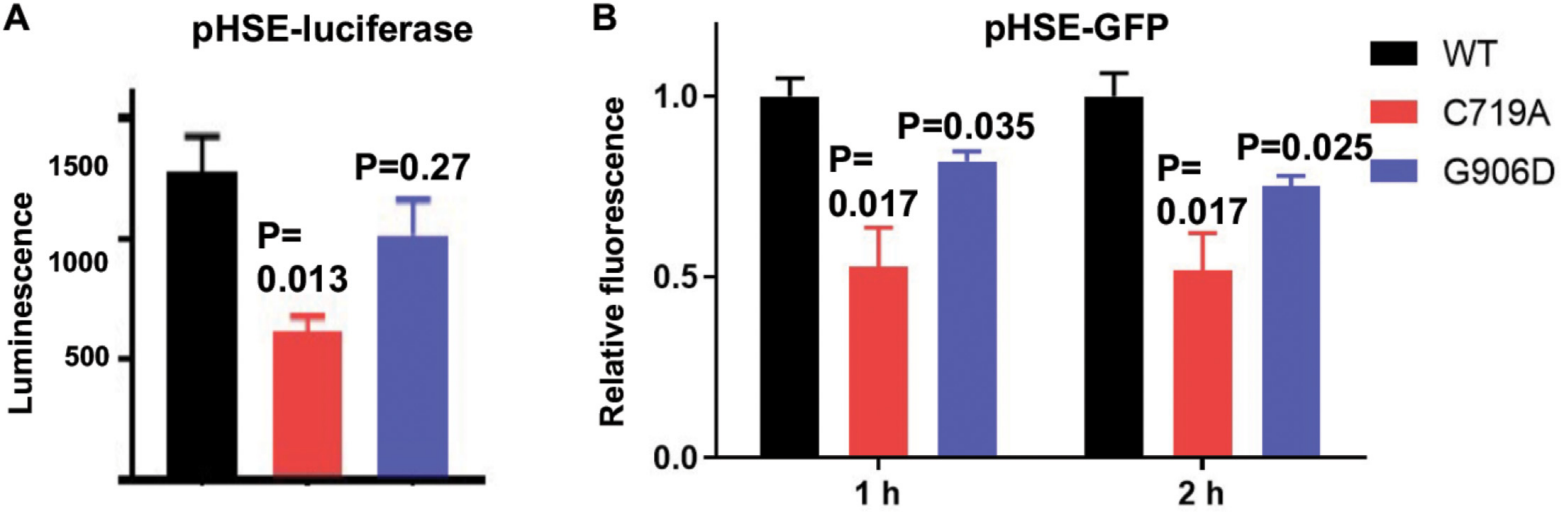
C719A AlaRS mutation attenuates the heatshock response. Both pHSE-luciferase (**A**) and pHSE-GFP (**B**) reporters show that the heatshock response is attenuated in the C719A mutant strain. These results are the average of at least three biological replicates. Error bars represent standard errors. The *P* value is determined using the unpaired *t* test.

The proteomics data revealed that the abundance of heatshock proteins was decreased in the proteome of the AlaRS C719A strain (Figure 4), prompting us to investigate the heatshock response in the editing and aminoacylation defective strains. Using both luciferase and green fluorescence protein (GFP) reporters under the control of the heatshock element (HSE) promoter (29,43), we found that the heatshock response was significantly reduced in the C719A strain compared with the WT (Figure 7). The heatshock response activates expression of chaperones to protect cells from protein denaturing conditions such as heat (44,45). We further tested protein aggregation by purifying aggregates and electrophoresis on SDS-PAGE gel, as well as using a HSP104-RFP (red fluorescence protein) reporter (30). The C719A mutation did not appear to substantially increase total protein aggregates or the number of aggregated foci (Supplementary Figure S9). In yeast, a ribosomal quality control (RQC) mechanism adds Ala and Thr tails to stalled peptides and target them for degradation (31). The RQC process requires *LTN1*, *RQC1* and *RQC2*, and contributes to protein quality control and heat resistance (31,43). Using a reporter for the RQC pathway (31), we showed that there was no difference in RQC among WT, C719A or G906D strains (Supplementary Figure S10), ruling out the role of RQC in our observed heat sensitivity of the AlaRS editing-defective strain.

### Extra carbon source partially rescues heat sensitivity caused by AlaRS editing deficiency

**Figure 8.**
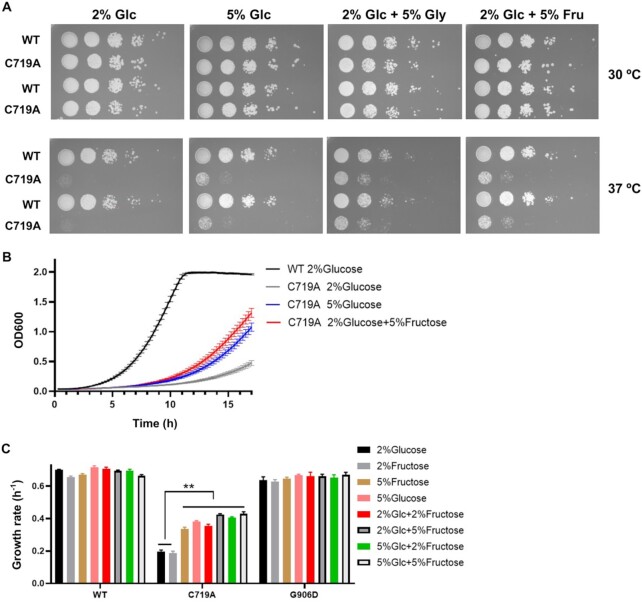
Additional carbon source improves growth of C719A AlaRS yeast variant at 37°C. (**A**) Yeast cells were grown to log phase and spotted on YPD agar plates with additional carbon sources with 10-fold dilutions. The plates were incubated at 30 or 37°C for 2 days before imaging. (**B**, **C**) Growth rates of yeast strains in YPD liquid culture with additional carbon sources at 37°C. These results are the average or representatives of at least three biological replicates. Error bars represent standard errors. The *P* value is determined using the unpaired *t* test. ** *P* < 0.01. Glc, glucose; Gly, glycerol; Fru, fructose.

The most significantly downregulated pathway in the C719A strain, as revealed by both RNA sequencing and proteomics analyses, is carbon metabolism (Figures 3 and 4). Impaired carbon metabolism is expected to decrease synthesis of amino acids. In support of this notion, we found that supplementing the glucose media with extra glycerol carbon source suppressed the GCN4 GAAC response in the C719A strain (Supplementary Figure S11). Interestingly, additional carbon sources also partially rescued the growth defect of C719A at 37°C (Figure 8), likely through increasing heatshock proteins and energy source to properly fold mistranslated proteins.

## DISCUSSION

**Figure 9.**
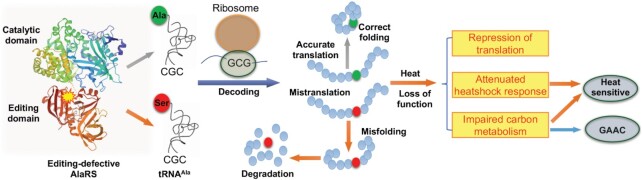
Model of cellular impact caused by AlaRS editing deficiency. AlaRS harbors catalytic (aminoacylation) and editing domains. Editing deficiency in AlaRS leads to accumulation of Ser-tRNA^Ala^ and Ser misincorporation at Ala codons. Ser mistranslation promotes misfolding, degradation and loss of function for some key regulatory proteins, resulting in defects in translation, heatshock response and carbon metabolism.

Decades of biochemical and structural studies have elucidated the mechanisms of editing by aminoacyl-tRNA synthetases and trans-editing factors (9,10,12,14,24,35–37,46–56). However, why editing is conserved throughout evolution in many aaRSs has been a perplexing question given that it is dispensable under normal growth conditions. Based on our systematic analyses in yeast, we propose the following model for the function of AlaRS editing (Figure 9): AlaRS uses a cis-editing domain to prevent accumulation of misacylated tRNA^Ala^, such as Ser-tRNA^Ala^; AlaRS editing deficiency causes misincorporation of Ser into Ala positions in the proteome, which leads to a cascade of misfolding and degradation events of key regulatory proteins; Heat stress exacerbates protein misfolding and results in loss of function for key factors in the carbon metabolism, heatshock response and protein synthesis pathways. Exactly which key regulatory proteins are affected by Ser misincorporation remain to be elucidated in future studies. As a result of impaired carbon metabolism and heatshock response, the GAAC pathway is activated to compensate for the amino acid biosynthesis deficiency, and the cells also become sensitive to heat stress. This is supported by our observations that extra carbon source suppresses the GAAC response and partially rescues the heat sensitivity of the editing-defective strain (Figure 8 and Supplementary Figure S8). The GAAC pathway is known as the integrated stress response (ISR) in mammals (57,58). A previous study reveals that a mutation in tyrosyl-tRNA synthetase (TyrRS) that leads to peripheral neuropathy activate ISR by localizing TyrRS in the nucleus (59). In future studies, it would be important to systematically characterize the role of disease-associated aaRS mutations in regulation of ISR, as well as the underlying mechanisms.

Editing deficiency of many aaRSs are well tolerated under normal growth conditions. Valyl- and phenylalanyl-tRNA synthetase editing-defective *E. coli* mutants grow as well as the WT unless their editing substrates accumulate in large amounts in the cells (19,60). ThrRS editing deficiency causes growth inhibition only when the heatshock proteases are absent (25). Recent studies suggest that aaRS editing could play critical roles under stressed conditions. For instance, lack of PheRS editing tempers the GAAC pathway in yeast due to accumulation of misacylated tRNA^Phe^ (61). Interestingly, AlaRS editing deficiency shows the opposite effect as PheRS and constitutively activates the GAAC response (Figures 3-5). We also show that ablating AlaRS editing attenuates the heatshock response and causes heat sensitivity (Figures 1, 4 and 7). In contrast, LeuRS editing deficiency as well as Ala misincorporation at Pro codons activate the heatshock response (20,62). It becomes increasingly clear that the editing function of different aaRSs may regulate distinct cellular response pathways. This is likely due to the nature of amino acid misincorporation resulting from different editing defects. Ser misincorporation at Ala codons due to AlaRS editing deficiency should exhibit distinct effects on the proteome as misincorporation at Leu codons. In support of this notion, whereas the tRNA^Ser^ variants (AGC and TGC) decoding Ala codons cause heat sensitivity, the tRNA^Ser^ variant (CAG) decoding Leu does not (Figure 2 and Supplementary Figure S4). Indeed, CTG Leu codons are ambiguously decoded as both Leu and Ser in *Candida albicans*, which promotes phenotypic diversity (63,64).

AlaRS is a unique aaRS in several aspects. While most aaRSs recognize the anticodons of tRNAs, AlaRS recognizes a single G3:U70 pair as the major tRNA determinants for both aminoacylation and editing (65–68). Editing defects in AlaRS also appear to be particularly detrimental, as evidenced by the severe neurological and cardiovascular disorders resulting from editing-defective AlaRS mutants in mice (21,22). Intriguingly, an AlaRS mutation (p.Tyr690Leufs*3) that lead to both aminoacylation and editing defects are found in human patients with progressive microcephaly and epileptic encephalopathy (23). The other AlaRS allele in these patients carry a mutation (G913D, equivalent to G906D in yeast) that decreases the aminoacylation efficiency. It is unclear how AlaRS editing deficiency contributes to the disease onset in the patients. We attempted to introduce both AlaRS mutations in yeast but were only able to obtain the G906D mutant strain, suggesting that a combination of severe aminoacylation and editing defects in AlaRS is lethal for yeast. This could be explained by loss of function resulting from AlaRS editing deficiency, which may destabilize the proteome and dysregulate key cellular pathways.

## CONCLUDING REMARKS

In summary, our work here reveals the critical function of AlaRS editing in maintaining normal metabolism and stress responses in yeast cells, which is distinct from other aaRSs. The physiological roles of translational fidelity may vary due to the different nature and severity of translational errors.

## DATA AVAILABILITY

The RNA sequencing data are deposited in Genome Expression Omnibus (GEO, accession GSE178694): https://www.ncbi.nlm.nih.gov/geo/query/acc.cgi?acc=GSE178694.

The mass spectrometry proteomics data have been deposited to the ProteomeXchange Consortium via the PRIDE (33) partner repository with the dataset identifier PXD026930.

## Supplementary Material

gkab766_Supplemental_Files

## Notes

Present address: Jiang Wu, RevoChem LLC, 16203 Park Row Suite 100, Houston, TX 77084, USA.
