# Supplementary material for: Impact of alanyl-tRNA synthetase editing deficiency in yeast
Source: Nucleic Acids Res. 2021 Sep 9;49(17):9953–64. doi: 10.1093/nar/gkab766 (PMC8464055; doi:10.1093/nar/gkab766)
Supplement: gkab766_Supplemental_Files [file gkab766_supplemental_files.zip › SI_Zhang et al.pdf]

Impact of Alanyl-tRNA Synthetase Editing Deficiency in Yeast

Hong Zhang[1], Jiang Wu[2], Zhihui Lyu[1], Jiqiang Ling[1]

[1]Department of Cell Biology and Molecular Genetics, The University of Maryland, College Park, MD 20742, USA
[2]Department of Microbiology and Molecular Genetics, McGovern Medical School, University of Texas Health Science Center, Houston, TX 77030, USA
*Correspondence should be addressed to:
Jiqiang Ling: +1 (301) 405-1035; Email: jling12@umd.edu

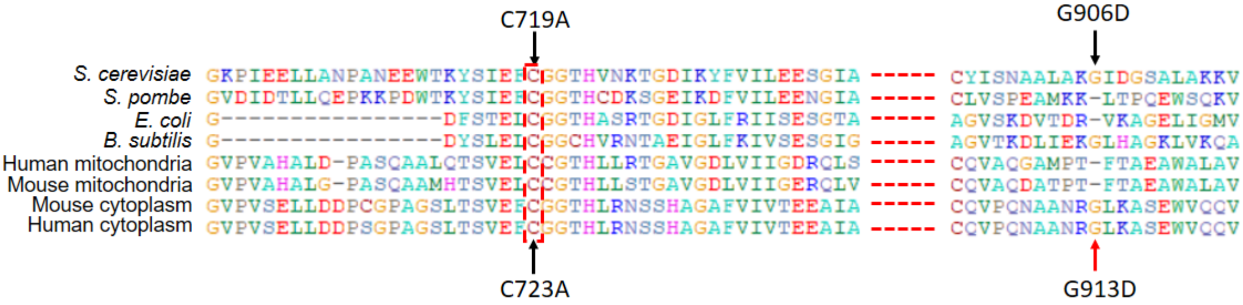

Figure S1. Amino acid sequence alignment of AlaRSs. The C719 and G906 residues of *S. cerevisiae* AlaRS are located in the editing domain and C-terminus, respectively. The equivalent residues in the human AlaRS are also indicated.

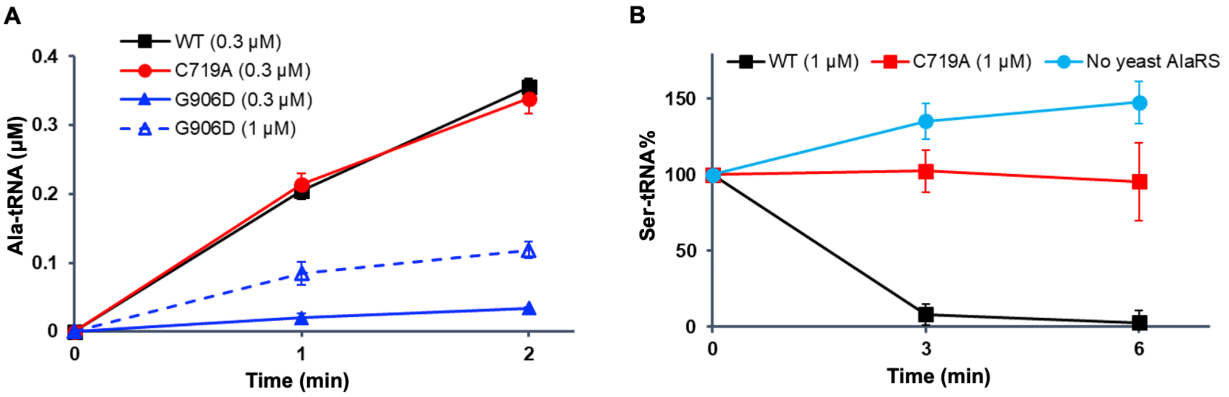

Figure S2. Aminoacylation and editing of yeast AlaRS variants. (A) Alanylation activity in the presence of 40 μM [^{14}C] alanine, 20 mg/ml total yeast tRNA, and indicated concentrations of yeast AlaRS variants at 37 ºC. (B) Editing activity of yeast AlaRS variants. 100 μM [^{14}C] serine and 20 mg/ml total yeast tRNA were preincubated with 3 μM human AlaRS C723A protein at 37 ºC to allow formation of Ser-tRNA^{Ala}. WT and C719A Yeast AlaRS variants were then added to the reaction separately, and the remaining Ser-tRNA over time was determined. Whereas WT yeast AlaRS efficiently hydrolyzes (edits) Ser-tRNA, the C719A mutant is defective in editing. These results are the average of at least three replicates. Error bars represent standard deviations.

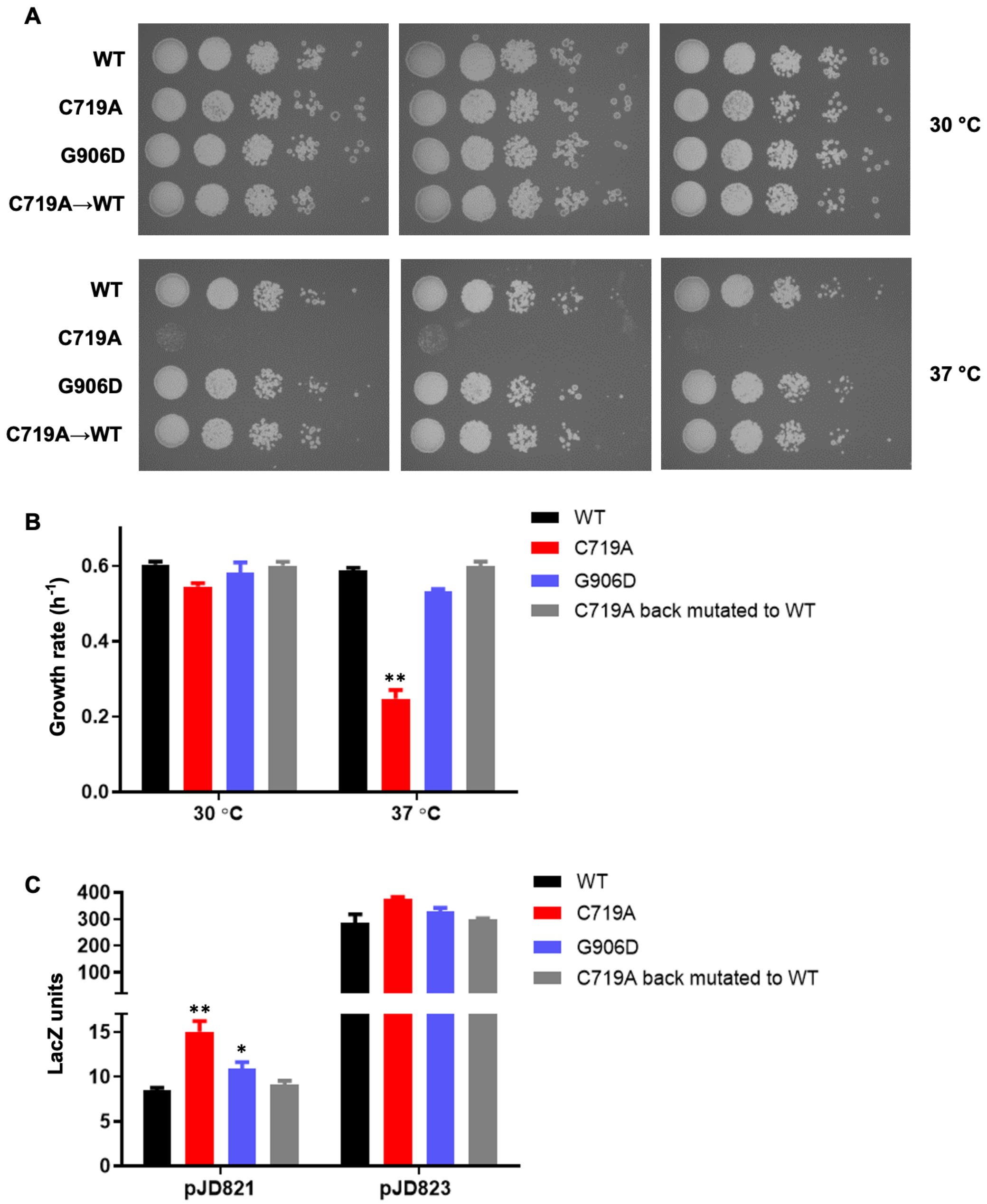

Figure S3. Independent verification of heat sensitivity and GCN4 GAAC activation phenotypes. Plasmid shuffling was carried out independently from the strains used in the main figures to generate new WT, C719A, and G906D AlaRS variant strains. In addition, the C719A mutation was reversed to the WT. The experiments were performed as Figures 1 and 5. The (A, B) heat sensitivity and (C) GCN4 activation phenotypes are consistent with Figures 1 and 5.

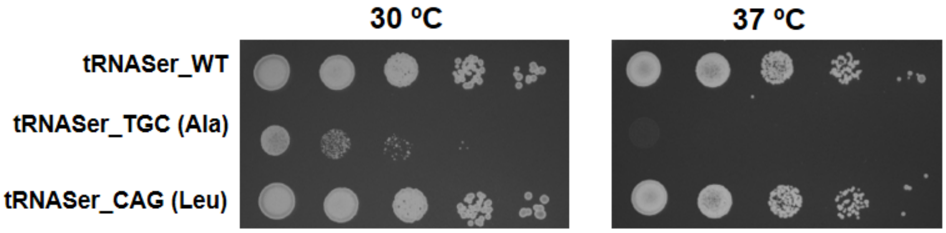

Figure S4. Growth of yeast strains expressing $tRNA^{Ser}$ variants reading Ala and Leu codons. WT yeast carrying pRS315 $tRNA^{Ser}$ variants were grown in SD - Leu medium to log phase and spotted on SD - Leu plates with 10-fold dilutions. The plates were incubated at 30 or 37 °C for 4 days before imaging. These results are the representatives of at least three biological replicates.

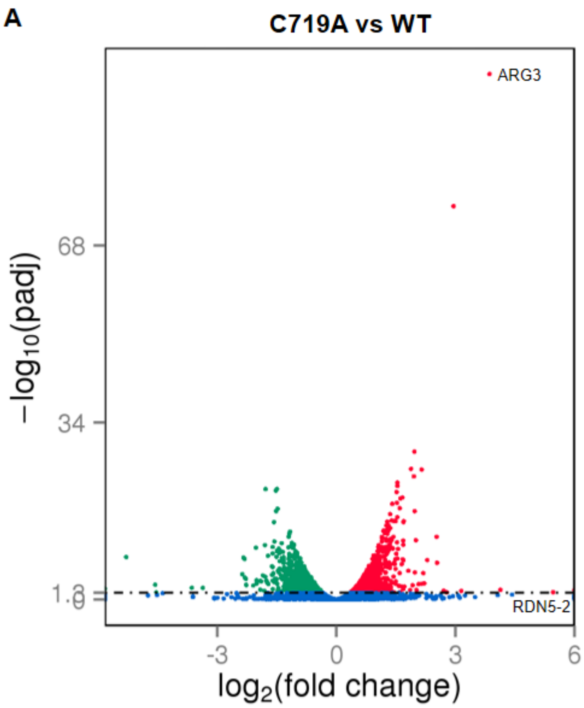

C719A vs WT upregulated pathways

Term	Q value
Ribosome	7.77E-16
Ribosome biogenesis in eukaryotes	2.10E-08
Pyrimidine metabolism	1.91E-06
Biosynthesis of amino acids	2.85E-06
RNA polymerase	0.00011
2-Oxocarboxylic acid metabolism	0.00018
Purine metabolism	0.00046
Metabolic pathways	0.00067
Valine, leucine and isoleucine biosynthesis	0.00409
Arginine biosynthesis	0.00857
Phenylalanine, tyrosine and tryptophan biosynthesis	0.02237
RNA transport	0.03831
Biosynthesis of secondary metabolites	0.04728

C719A vs WT downregulated pathways

Term	Q value
Glycolysis / Gluconeogenesis	0.00254
Starch and sucrose metabolism	0.02044

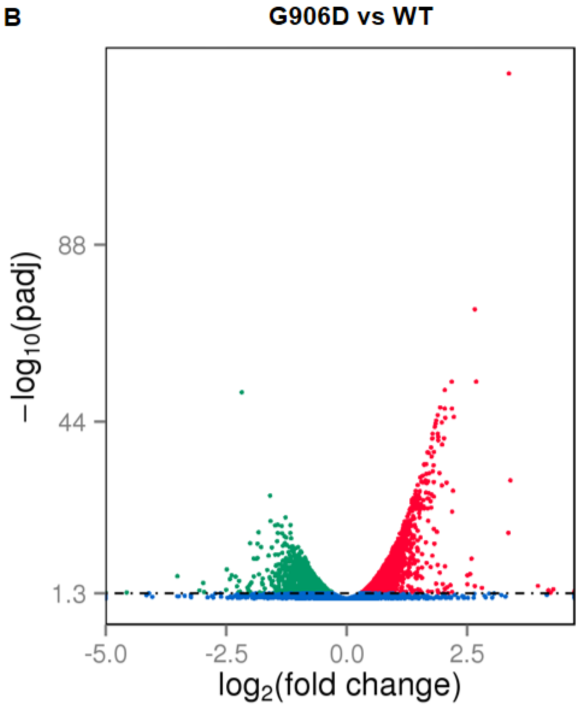

G906D vs WT upregulated pathways

Term	Q value
Ribosome	3.22E-20
Ribosome biogenesis in eukaryotes	6.54E-06
Biosynthesis of amino acids	8.87E-05
Metabolic pathways	0.0004
Pyrimidine metabolism	0.00048
RNA polymerase	0.00061
Purine metabolism	0.00119
RNA transport	0.00351
RNA degradation	0.02528

Figure S5. RNA sequencing comparison of yeast variants before heat stress. Yeast cells were grown in YPD at 30 °C to log phase and total RNA was prepared. (A) Comparing C719A mutant with the WT. (B) Comparing the G906D mutant with the WT. Three biological replicates were tested for each strain at each condition.

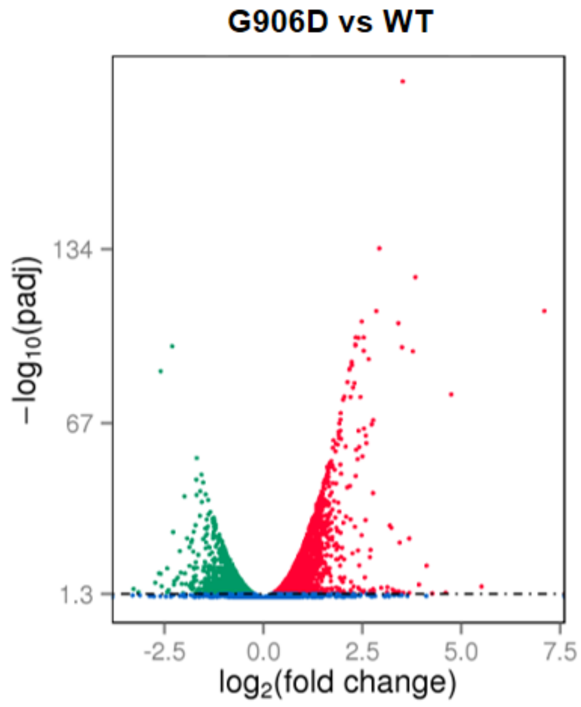

G906D vs WT upregulated pathways

Term	Q value
Ribosome	9.01E-15
Biosynthesis of amino acids	1.71E-07
Ribosome biogenesis in eukaryotes	2.74E-05
RNA polymerase	0.00169
Pyrimidine metabolism	0.00184
Biosynthesis of antibiotics	0.00309
Metabolic pathways	0.00309
Proteasome	0.0047
Purine metabolism	0.00505
Biosynthesis of secondary metabolites	0.01255
2-Oxocarboxylic acid metabolism	0.01259
Phenylalanine, tyrosine and tryptophan biosynthesis	0.01739
RNA transport	0.0213

Figure S6. RNA sequencing comparison of G906D and WT yeasts upon heat stress. Yeast cells were grown in YPD at 30 °C to log phase and shifted to 37 °C for 2 hours before total RNA was prepared. Three biological replicates were tested for each strain at each condition.

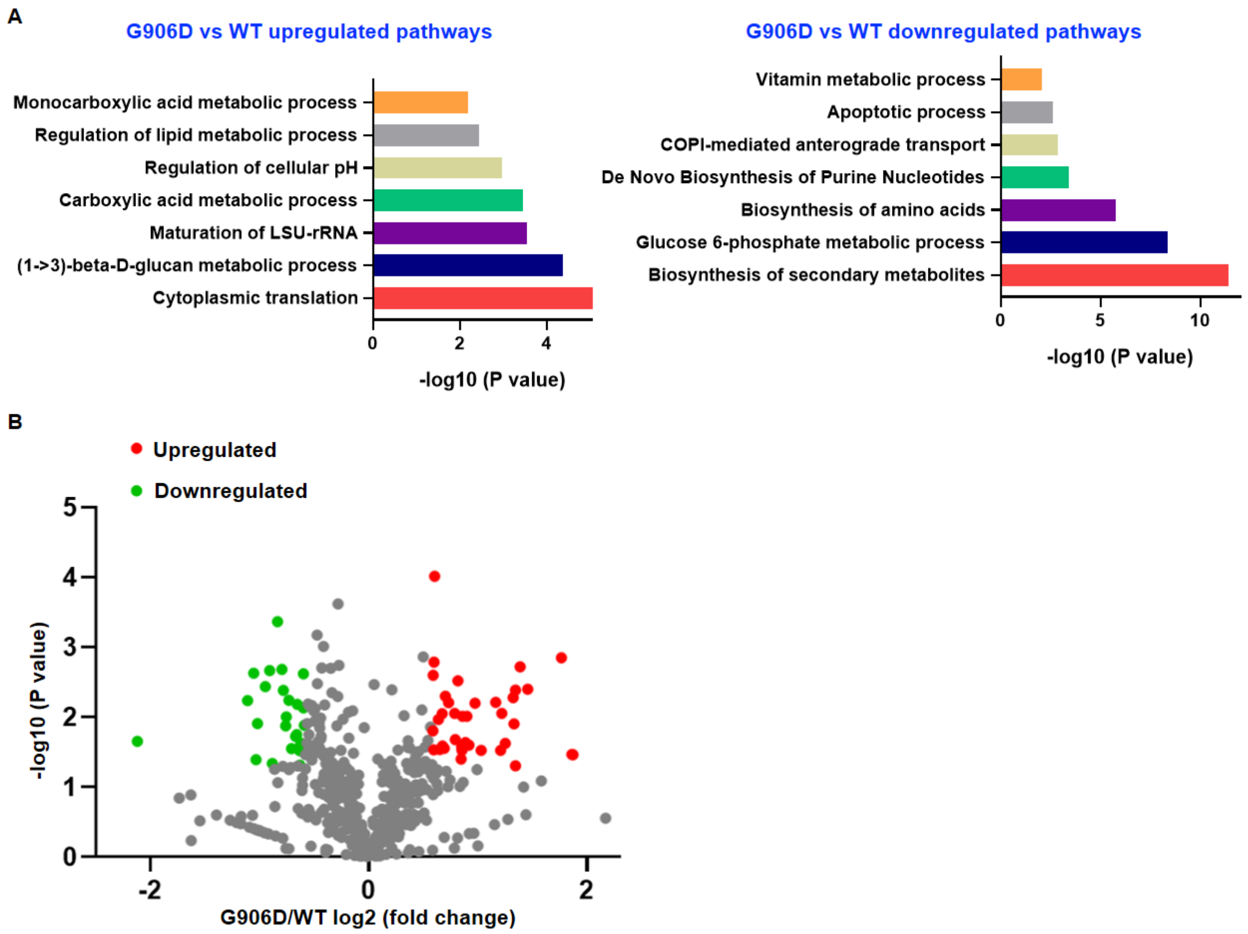

Figure S7. Quantitative proteomics of G906D yeast compared to WT upon heat stress. Yeast cells were grown in YPD at 30 °C to log phase and shifted to 37 °C for 2 hours before rapid freezing and protein extraction. (A) Significantly enriched pathways in the G906D mutant compared with the WT. (B) Volcano plot comparing G906D and WT. Three biological replicates were tested for each strain at each condition.

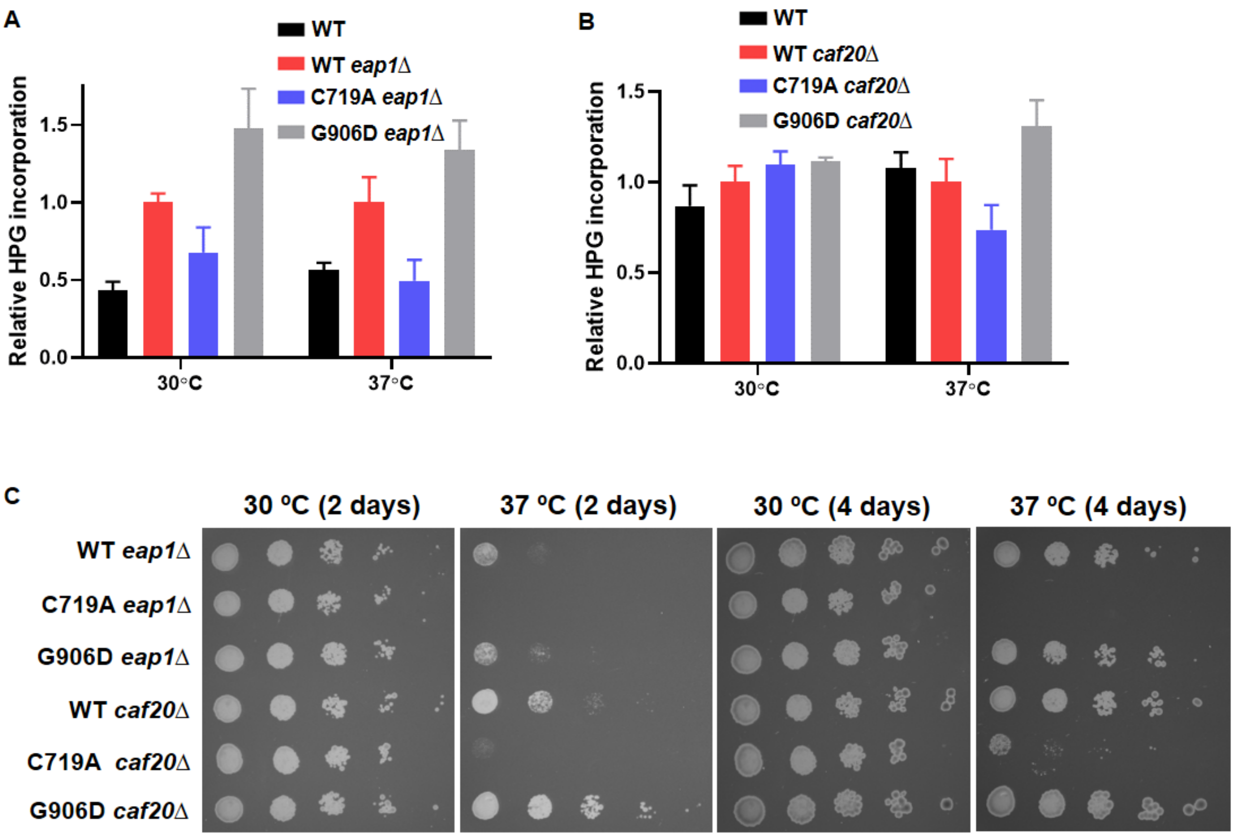

Figure S8. Effects of deleting *eap1* and *caf20* on protein synthesis and growth. (A, B) HPG incorporation was determined as in Figure 6. (C) Yeast cells were grown to log phase and spotted on YPD agar plates with 10-fold dilutions. The plates were incubated at 30 or 37 °C for 2 or 4 days before imaging. These results are the average or representatives of at least three biological replicates. Error bars represent standard errors.

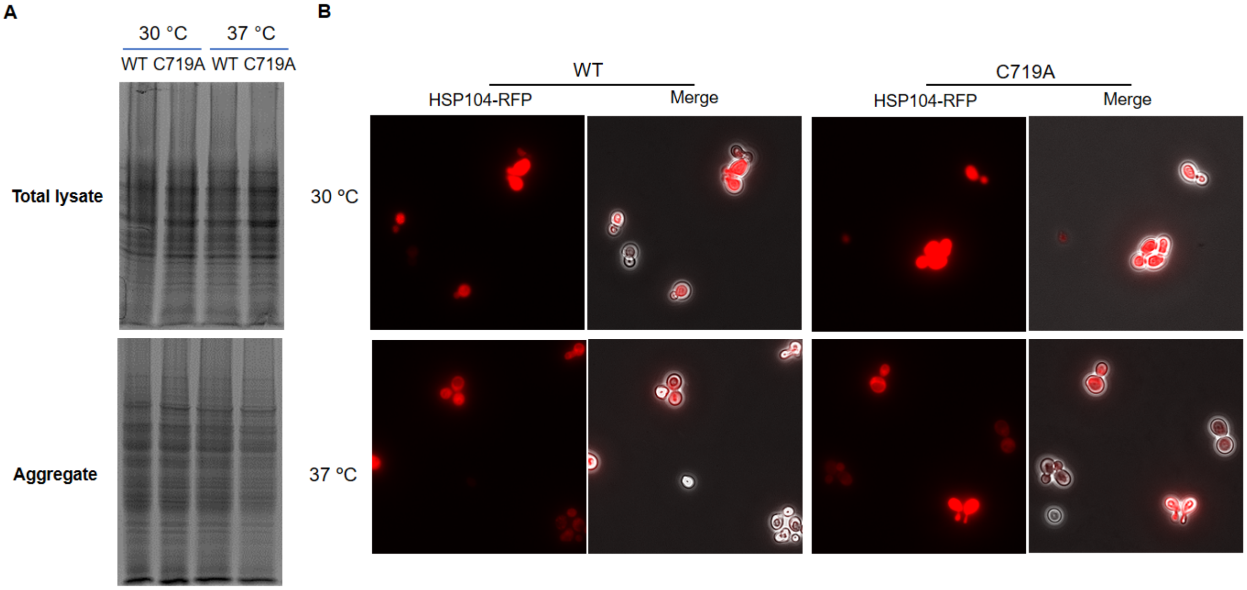

Figure S9. C719A AlaRS mutation does not induce protein aggregation. (A) SDS-PAGE gel analysis of total lysate and aggregates in the WT and C719A yeast strains grown at 30 or 37 °C. (B) Fluorescence and phase-contrast imaging of WT and C719A yeast strains carrying pHSP104-RFP grown at 30 or 37 °C.

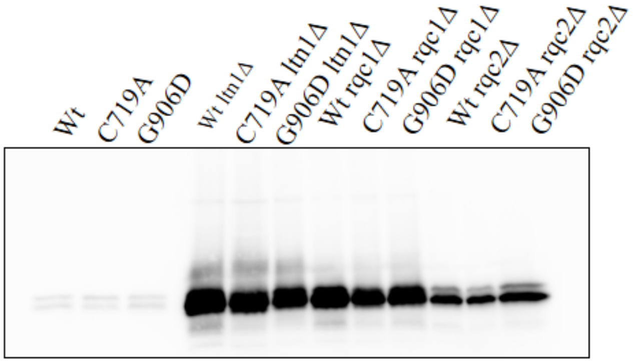

Figure S10. Ribosome quality control in yeast variants. Strains carrying pTDH3-GFP-R12-RFP were grown in SD - Ura at 30 °C. Western blot against GFP was performed to detect truncated and full-length reporters. Ribsomes stall at tandem arginine (R12) codons between GFP and RFP genes. Active RQC results in degradation of GFP. Deleting the RQC genes *ltn1*, *rqc1* or *rqc2* results in similar accumulation of GFP in the WT, C719A, and G906D strains, indicating that C719A and G906D mutations do not significantly affect the RQC pathway. This result is the representative of three biological replicates.

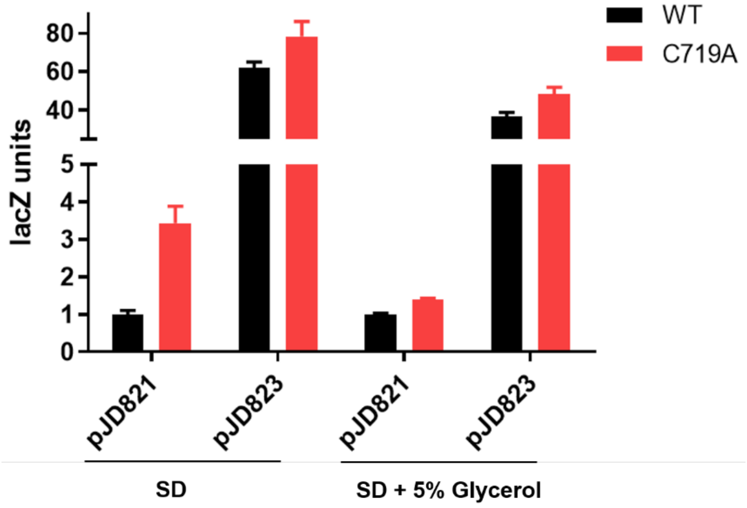

Figure S11. Additional carbon source represses *GCN4* activation in the C719A strain. LacZ reporter assay for *GCN4* pathway was determined as in Figure 5. The results are the average of three biological replicates.

Table S1. Differential gene expression in WT, C719A, and G906D yeast strains revealed by RNA sequencing.

Table S2. Proteomics of WT, C719A, and G906D yeast strains.

Table S3. Pathway enrichment of differentially expressed proteins.

Table S4. Strains and plasmids used in this study.

Strain or Plasmid	Relevant properties	Sources
**Strain**		
E. coli DH5α	Used for plasmids construction	Ling lab
Yeast WT	BY4741	This study
C719A	BY4741, AlaRS point mutation C719A	This study
G906D	BY4741, AlaRS point mutation G906D	This study
WT *gcn2*Δ	*GCN2* was replaced by *URA3*	This study
C719A *gcn2*Δ	*GCN2* was replaced by *URA3*	This study
G906D *gcn2*Δ	*GCN2* was replaced by *URA3*	This study
WT *gcn4*Δ	*GCN4* was replaced by *URA3*	This study
C719A *gcn4*Δ	*GCN4* was replaced by *URA3*	This study
G906D *gcn4*Δ	*GCN4* was replaced by *URA3*	This study
WT *alaX*Δ	*ALAX* was replaced by *URA3*	This study
C719A *alaX*Δ	*ALAX* was replaced by *URA3*	This study
G906D *alaX*Δ	*ALAX* was replaced by *URA3*	This study
WT *ltn1*Δ	*LTN1* was replaced by *HIS3*	This study
C719A *ltn1*Δ	*LTN1* was replaced by *HIS3*	This study
G906D *ltn1*Δ	*LTN1* was replaced by *HIS3*	This study
WT *rqc1*Δ	*RQC1* was replaced by *HIS3*	This study
C719A *rqc1*Δ	*RQC1* was replaced by *HIS3*	This study
G906D *rqc1*Δ	*RQC1* was replaced by *HIS3*	This study
WT *rqc2*Δ	*RQC2* was replaced by *HIS3*	This study
C719A *rqc2*Δ	*RQC2* was replaced by *HIS3*	This study
G906D *rqc2*Δ	*RQC2* was replaced by *HIS3*	This study
WT *eap1*Δ	*EAP1* was replaced by *URA3*	This study
C719A *eap1*Δ	*EAP1* was replaced by *URA3*	This study
G906D *eap1*Δ	*EAP1* was replaced by *URA3*	This study
WT *caf20*Δ	*CAF20* was replaced by *URA3*	This study

C719A *caf20*Δ	*CAF20* was replaced by *URA3*	This study
G906D *caf20*Δ	*CAF20* was replaced by *URA3*	This study
**Plasmid**		
pRS315	Expression vector for yeast, *LEU2*	Ling lab
pRS316	Expression vector for yeast, *URA3*	Ling lab
pJD821	P*gcn4*-*lacZ* reporter, *URA3*	Dinman lab
pJD822	P*gcn4*-*lacZ* reporter, *URA3*	Dinman lab
pJD823	P*gcn4*-*lacZ* reporter, *URA3*	Dinman lab
pJD1212	Cherry-Venus expression, *URA3*	Dinman lab
pHSE-lucCP$^+$	Heat-induced expression of luciferase	Morano lab
pGre2-lucCP$^+$	Salt-induced expression of luciferase	Morano lab
p4HSE-GFP	Heat-induced expression of GFP	Morano lab
pTDH3-GFP-R12-RFP	Ribosome quality control reporter	Brandman lab
pHSP104-RFP	Aggregation reporter	Megeney lab
pJL-*bla*	β-lactamase expression	This study
pJL-*bla*-S68A-GCC	β-lactamase S68A expression	This study
pRS316AlaRS-WT	AlaRS expression, *URA3*	This study
pRS315AlaRS-WT	AlaRS expression, *LEU2*	This study
pRS315AlaRS-C719A	AlaRS C719A expression, *LEU2*	This study
pRS315AlaRS-G906D	AlaRS G906D expression, *LEU2*	This study
pRS315tRNASer_AGA(WT)	tRNASer_AGA(WT) expression, *LEU2*	This study
pRS315tRNASer_AGC(Ala)	tRNASer_AGC(Ala) expression, *LEU2*	This study
pRS315tRNASer_TGC(Ala)	tRNASer_TGC(Ala) expression, *LEU2*	This study
pRS315tRNASer_CAG(Leu)	tRNASer_CAG(Leu) expression, LEU2	This study

Table S5. Oligos used in this study.

Oligo name	Sequences (5'-3')	Description
Optimized *bla* gene for yeast	TAGAACTAGTATGGAAACTTTGGTCAAGGTTAAGGATGCTGAAGACCAATTAGGTGCCAGAGTTGGTTACATTGAACTAGATTTGAATTCAGGTAAGATCTTGGAATCTTTCAGACCAGAAGAAAGATTCCCAATGATGTCCACTTTCAAGGTCTTGTTGTGTGGTGCTGTCTTGTCCAGAGTCGATGCCGGTCAAGAACAATTGGGTAGAAGAATTCACTATTCTCAAAACGACTTGGTTGAGTACTCTCCAGTCACTGAAAAGCATTTGACTGATGGTATGACTGTTAGAGAACTCTGTTCTGCCGCCATCACCATGTCTGACAACACTGCTGCCAACTTGTTATTGACCACCATTGGTGGTCCAAAGGAATTGACCGCTTTCTTGCACAACATGGGTGACCACGTTACCAGATTGGACAGATGGGAACCAGAATTGAACGAAGCTATCCCAAACGATGAAAGAGATACTACAATGCCAGCTGCTATGGCTACTACCTTGAGAAAGTTGTTAACCGGTGAATTATTGACTTTGGCTTCCCGTCAACAATTGATTGACTGGATGGAAGCTGACAAAGTTGCTGGTCCATTGCTAAGATCTGCTTTGCCTGCTGGTTGGTTTATTGCTGATAAATCCGGCGCTGGTGAACGTGGTAGTAGAGGTATCATCGCCGCTTTGGGTCCAGACGGTAAGCCATCTCGTATTGTTGTCATCTACACCACTGGTTCCCAAGCTACCATGGACGAAAGAAACAGACAAATCGCTGAAATTGGTGCCTCTTTGATCAAGCACTGGTAGAAGCTTATCG	Amplification of β-lactamase gene, and the amplicon was constructed into digested pJD1212 to obtain the vector pJL-*bla*
Bla-F	CTTAGTTTCGACGGATTCTAGAACTAGTATGGAAACTTTGGTCA	
Bla-R	ACTCGAGGTAGACGGTATCGATAAGCTTCTACCAGTGCTTGATC	
S68A(GCC)-mutation-UF	TCACTAAAGGGAACAAAAGCTGGAGCTCAGTTTATCATTATCAATACTCGC	UF/UR: amplification of the upstream codon region of β-lactamase; DF/DR amplification of the downstream codon region of
S68A(GCC)-mutation-UR	GGCCATCATTGGGAATCTTTCTTCTGGTCTGAAAGA	

S68A(GCC)-mutation-DF	AGAAAGATTCCCAATGATGGCCACTTTCAAGGTCTTGTTGTG	β-lactamase; two fragments were assembled into digested pJD1212 to obtain pJL-bla-S68A-GCC.
S68A(GCC)-mutation-DR	ACTCGAGGTAGACGGTATCGATAAGCTTCTACCAGTGCTTGATCAAAG	
S68A(GCG)-mutation-R	TCGCCATCATTGGGAATCTTTCTTCTGGTCTGAAAGA	
tRNASer_AGA(WT)-F	GCGGTGGCGGCCGCTCTAGAACTAGTAGCCAATTCTCAGCAACAAATC	Amplification of tRNASer_AGA(WT) and the amplicon was infused into pRS315.
tRNASer_AGA(WT)-R	TCGAGGTCGACGGTATCGATAAGCTTGACGTATGCTTTCTTTAACGTTC	
tRNASer_AGC(Ala)-UF	GCGGTGGCGGCCGCTCTAGAACTAGTAGCCAATTCTCAGCAACAAATC	Amplification of tRNASer_AGC(Ala) and the amplicon was infused into pRS315.
tRNASer_AGC(Ala)-UR	CGGGCAAAGCCCAAAAGATTGCTAATCTTTC	
tRNASer_AGC(Ala)-DF	AATCTTTTGGGCTTTGCCCGC	
tRNASer_AGC(Ala)-DR	TCGAGGTCGACGGTATCGATAAGCTTGACGTATGCTTTCTTTAACGTTC	
tRNASer_TGC(Ala)-UF	GCGGTGGCGGCCGCTCTAGAACTAGTAGCCAATTCTCAGCAACAAATC	Amplification of tRNASer_TGC(Ala) and the amplicon was infused into pRS315.
tRNASer_TGC(Ala)-UR	CGGGCAAAGCCCAAAAGATTGCAAATCTTTC	
tRNASer_TGC(Ala)-DF	AATCTTTTGGGCTTTGCCCGC	
tRNASer_TGC(Ala)-DR	TCGAGGTCGACGGTATCGATAAGCTTGACGTATGCTTTCTTTAACGTTC	
tRNASer_CAG(Leu)-UF	GCGGTGGCGGCCGCTCTAGAACTAGTAGCCAATTCTCAGCAACAAATC	Amplification of tRNASer_CAG(Leu) and the amplicon was infused into pRS315.
tRNASer_CAG(Leu)-UR	TTCTGAATCTTTCGCCTTAACCACTCGGCCAAGTTGCC	
tRNASer_CAG(Leu)-DF	GTGGTTAAGGCGAAAGATTCAGAATCTTTTGGGCTTTGCCCG	
tRNASer_CAG(Leu)-DR	TCGAGGTCGACGGTATCGATAAGCTTGACGTATGCTTTCTTTAACGTTC	

Gcn2-KO-F	GATTTTTTTTTTCAATAATTTTCCGTTCCCCTTAACACATACTATGTATAAcgcttttcaattcaattcat	Amplification of *URA3*, and the donor DNA was transformed into WT/C719A/G906D to obtain *gcn2*Δ.
Gcn2-KO-R	TACTTTACCTTTAACTGATGCGTTATAGCGCCGCACAGATCTTTAAAGGCcctgatgcggtattttct	
Gcn2-KO-OVF	AATCTTGACGTGCAAGGGCC	To verify and select *gcn2*Δ.
Gcn2-KO-OVR	ACCTACCCCCTTTACAACGGT	
Gcn2-IVR	TTTCGCTCGAGGAAGTCGAG	
Gcn4-KO-UF	ACTCACAACACAGGTTACTCT	amplification of *URA3*, and the donor DNA was transformed into WT/C719A/G906D to obtain *gcn4*Δ.
Gcn4-KO-UR	CTTGAGCAGACAAATTGGTAAAC	
URA3-F	TTTACCAATTTGTCTGCTCAAGcacgcttttcaattcaattcat	
URA3-R	cctgatgcggtattttct	
Gcn4-KO-DF	CGAACGCTGATTTCATTTACC	
Gcn4-KO-DR	GAAGCTGAAATGCAAGGATTG	
Gcn4-KO-OVF	TCCGTAACGGTTACCTTTCTGT	To verify and select *gcn4*Δ.
Gcn4-KO-OVR	CGTAATGGCAACGCGTCTGA	
Gcn4-IVR	GCAACTTTCTCGCACGAG	
alaX-KO-F	GTGACGCATATCGACGAAAGCACACAACAGCGTCAAAAATTGATTAAAAGGTAAGTTATCcgcttttcaattcaattcat	amplification of *URA3*, and the donor DNA was transformed into WT/C719A/G906D to obtain *alaX*Δ.
alaX-KO-R	TCACGATAAATACCTACCATTATGTATACAACAAATCTGCGTATACAAATGGCACATTTCcctgatgcggtattttct	
alaX-KO-OVF	AAGAACCAACCACCCATCAT	To verify and select *alaX*Δ.
alaX-KO-OVR	GCGTTTGCATGTTCGAAGTTG	
URA3-IVR	ccttggtggtacgaacatcc	
Ltn1-KO-UF	CCAACACGACAATCATGAGTA	amplification of *HIS3*, and the donor DNA was transformed into WT/C719A/G906D to obtain *ltn1*Δ.
Ltn1-KO-UR	TAAGAACCAACTATTGCTTGAAC	
HIS3-F	TTCAAGCAATAGTTGGTTCTTAgagcttggtgagcgctag	
HIS3-R	cagaatgacacgtatagaatgatg	

Ltn1-KO-DF	atcattctatacgtgtcattctgCGAGGTTGTATTGAATGATAAGG	
Ltn1-KO-DR	ACTGATAAAGTGTATGGCCC	
Ltn1-KO-VF	CATCATTTGCGTCATCTTC	To verify and select *ltn1*Δ.
Ltn1-KO-VR	AGATGAACTCTGAGACAAAC	
Rqc1-KO-UF	GGAAAATGCAAAGAGCAGTT	Amplification of *HIS3*, and the donor DNA was transformed into WT/C719A/G906D to obtain *rqc1*Δ.
Rqc1-KO-UR	AGAGCTCATGTTCGAGTACT	
HIS3-F	AGTACTCGAACATGAGCTCTgagcttggtgagcgctag	
HIS3-R	cagaatgacacgtatagaatgatg	
Rqc1-KO-DF	atcattctatacgtgtcattctgAGAGTCAAATGAAAATGAGGGT	
Rqc1-KO-DR	ACAATGCAATACCAGGCA	
Rqc1-KO-VF	CGTCAGAAGATCATACCT	To verify and select *rqc1*Δ.
Rqc1-KO-VR	GACGCTAATCTTTGGAATG	
Rqc2-KO-UF	AGGATGATAGAAAGTCTCCG	Amplification of *HIS3*, and the donor DNA was transformed into WT/C719A/G906D to obtain *rqc2*Δ.
Rqc2-KO-UR	AATCTCTTGCCTGCCTGA	
HIS3-F	AGTCAGGCAGGCAAGAGATTgagcttggtgagcgctag	
HIS3-R	cagaatgacacgtatagaatgatg	
Rqc2-KO-DF	atcattctatacgtgtcattctgAAATCCACCAAGAACGGT	
Rqc2-KO-DR	AGCAAGGATTTTCAGCAATC	
Rqc2-KO-VF	GGCTTTCCAGGATAGCTA	To verify and select *rqc2*Δ.
Rqc2-KO-VR	CGAGGATCATTTAGTCCTTTA	
Eap1-KO-F	TGAATGTTTCAGTCTCTAAGCTACACAAAGAACTAGCATAGTAGGACTTTAACCCATACAcgcttttcaattcaattcat	Amplification of *URA3*, and the donor DNA was transformed into WT/C719A/G906D to obtain *eap1*Δ.
Eap1-KO-R	TAGATAGTATGTTCACTGTGTTTTTGTCGTTTCCTGTCAAGTACTCGCTCGTTACGCATCcctgatgcggtattttct	
Eap1-KO-OVF	GCAGCTAGTCATGTAATGGCAC	To verify and select *eap1*Δ.

Eap1-KO-OVR	AGTTCCGAGCGTGACGTTACTA	
URA3-IVR	ccttggtggtacgaacatcc	
Caf20-KO-F	AGACATTATTTGAGCTGTAACCTGAATATAGGATTAAGAACTTTTATTTAATTTCACGACcgcttttcaattcaattcat	Amplification of *URA3*, and the donor DNA was transformed into WT/C719A/G906D to obtain *caf20*Δ.
Caf20-KO-R	TTATTCAAAATTTATACATAGTACACGTACAGCTGCCTGAAGAAAGCATGAGCGAATGACcctgatgcggtattttct	
Caf20-KO-OVF	GTGGAATGGCAAGCAGTTCACT	To verify and select *caf20*Δ.
Caf20-KO-OVR	GTGCGTACGGCTCATGGTAGA	
URA3-IVR	ccttggtggtacgaacatcc	
